# Evaluation of the efficacy of the antimicrobial peptide HJH-3 in chickens infected with *Salmonella* Pullorum

**DOI:** 10.3389/fmicb.2023.1102789

**Published:** 2023-01-24

**Authors:** Yanzhao Xu, Qing Wang, Mengmeng Dong, Huishuai Song, Bolin Hang, Yawei Sun, Huihui Zhang, Jianhe Hu

**Affiliations:** College of Animal Science and Veterinary Medicine, Henan Institute of Science and Technology, Xinxiang, China

**Keywords:** antibiotic substitutes, antimicrobial peptide (AMP), HJH-3, *Salmonella* Pullorum, application effectiveness evaluation

## Abstract

As a promising substitute for antibiotics, increasing attention has been given to the clinical application of antimicrobial peptides (AMPs). In this study, the mode of action of the HJH-3 against *Salmonella* Pullorum was investigated. The structure and properties of HJH-3 were examined *in silico*, and minimum inhibitory concentrations (MICs) were determined to evaluate its antimicrobial spectrum. The time-kill kinetics of HJH-3 was determined. The hemolytic activity of HJH-3 was determined by measuring the hemoglobin ultraviolet absorption value, and the cytotoxicity was determined using a CCK-8 kit. The protective effect of HJH-3 on chickens infected with *S*. Pullorum was evaluated *in vivo*. The results demonstrated that HJH-3 exhibited strong antibacterial activity against Gram-negative pathogens at MIC values of 1.5625–25 μg/mL and against Gram-positive pathogens at MIC values of 25–50 μg/mL. HJH-3 also showed activity against the *Candida albicans* (100 μg/mL) and *Bacillus subtilis* (6.25–12.5 μg/mL). HJH-3 at 100 μg/mL completely killed *S.* Pullorum after co-incubation for 6 h. Likewise, the hemolysis rate of CRBCs treated with 100 μg/mL HJH-3 (7.31%) was lower than that of CRBCs treated with 100 μg/mL pexiganan (40.43%). Although the hemolysis rate of CRBCs treated with 400 μg/mL HJH-3 was increased to 13.37%, it was much lower than that of 400 μg/mL pexiganan (57.27%). In regards to cytotoxicity, HJH-3 had almost no-effect on the CEF proliferation, pexiganan decreased CEFs proliferation from 56.93 to 31.00% when increasing the concentration from 50 to 200 μg/mL. In a chicken infection model, the results showed that the antibiotic prevention and HJH-3 prevention groups exhibited the best treatment effect, with the chickens being protected from the lethal dose of *S.* Pullorum, a decreased number of bacteria in the blood and spleen, and less pathological changes in intestinal segments. The prevention of infection by HJH-3 was similar to that by Ampicillin; the effect of treatment after infection was lower than that of treatment before infection, and the survival rate of infected chicks treated with HJH-3 was 70%, which was still higher than that of the infected chickens. These results suggest that HJH-3 has good clinical application potential and can be used as a substitute for antibiotics for the prevention and treatment of *S*. Pullorum infection.

## Introduction

Antibiotics, the standard treatment to combat bacterial infections, are the mainstay of modern medicine and are integral to global health security. The use of antibiotics, the standard treatment for most bacterial infections, is one of the mainstays of the modern medicine. Although antibiotics are essential to ensure global health, their overuse and misuse are associated to the arise of “multidrug-resistant strains” (Robles-Jimenez et al., 2021; Ghimpeteanu et al., 2022). Although many kinds of “new antimicrobials,” such as second-, third-, and fourth-generation penicillins, have been discovered and developed successively in humans, researchers have not developed new antibiotics of real significance, and the continuous emergence of drug-resistant bacteria and the problem of antibiotic residues seriously threaten human health (Dutescu and Hillier, 2021; Ezure et al., 2022). Bacterial antimicrobial resistance has emerged as one of the leading public health threats of the 21st century. Therefore, there is an urgent need for new drugs with strong antibacterial effects, good safety, low propensity for resistance development and low residual effects (Safir et al., 2020; Bailly and Vergoten, 2021).

Antimicrobial peptides (AMPs), promising substitutes for antibiotics, are found in the tissues and cells of living organisms and are important molecules of the innate immune system. AMPs have broad-spectrum antibacterial activity, and their antibacterial mechanisms are different from those of conventional antibiotics (Wang et al., 2016). Most AMPs act on bacterial cell membranes directly to exert their antibacterial effects. Because the membrane structures of bacteria are relatively stable, resistance to AMPs rarely develops. In addition, the multi-targeted effects of AMPs are also characteristic of their broad antibacterial activity against drug-resistant strains (Zhu et al., 2022). Although most AMPs are only effective as topical agents and their antimicrobial activities are less potent than those of antibiotics, they have bright prospects as substitutes for antibiotics. However, most natural AMPs present a certain degree of cytotoxicity in addition to their antibacterial activity, which limits their direct application in medicine (Li et al., 2020). Modification of natural AMPs to obtain analogs with strong antimicrobial activity and low cytotoxicity has become a hotspot of AMP research (McMillan and Coombs, 2020; Kazemzadeh-Narbat et al., 2021).

Pullorum disease (PD) caused by *S.* Pullorum is a serious threat to the health of chickens worldwide. *S.* Pullorum is also a source of Salmonella contamination in poultry meat and eggs, caused by *S.* Pullorum which poses a threat to public health (Barrow and Freitas Neto, 2011; Li et al., 2019; Ding et al., 2021; Lv et al., 2022). Antibiotic therapy is still being used for PD in some countries. Although chemotherapeutic agents have been found to be effective at reducing mortality, they are not able to eliminate infection. Research results showed that the rates of Salmonella resistance to at least one antibiotic were 96, 98, and 56% in broilers, turkeys, and layers, respectively (Li et al., 2022). Therefore, it is necessary and urgent to find new antibacterial compounds to treat invasive salmonellosis (Drauch et al., 2020; Laure et al., 2021).

In our previous experiments, HJH-3 exhibited good antibacterial activity against *S.* Pullorum. It can directly enter the cell through the cell membrane and exert an antimicrobial effect (Zhang et al., 2015; Wang et al., 2019). In this study, chickens infected with *S.* Pullorum were used as the model to evaluate the clinical effect of HJH-3 *in vivo*. In addition, physical and chemical properties, the antibacterial spectrum, antibacterial kinetics, hemolysis activity, and cytotoxicity of HJH-3 were also determined.

## Materials and methods

### AMPs, bacterial strains and experimental materials

HJH-3 (Sequence: VNFKLLSHSLLVTLRSHL) was synthesized by Gill Biochemical Co., Ltd., Shanghai, China. Pexiganan (C122H210N32O22, MW 2477.17) was bought from Nanjing Peptide Biotechnology Co., Ltd., Nanjing, China. *S.* Pullorum (CVCC 533), *S.* Choleraesuis (CVCC 3776), *S.* Typhimurium (CVCC 541), *Escherichia coli* (*E. coli*, CVCC2059/1568), *Staphylococcus aureus* (*S.* Aureus CVCC 6538), and *Pseudomonas aeruginosa* (CVCC2087) were purchased from the China Institute of Veterinary Drug Control (CVCC, Beijing, China). *E. coli* (ATCC^®^ 25922™), *S.* Aureus (ATCC^®^25923™), *Candida albicans* (*C.* Albicans (ATCC^®^90029™) were purchased from the American Type Culture Collection (ATCC, Manassas, VA, USA). *B.* Pumilus (CMCC 63202), and *Bacillus* Subtilis (CMCC63501/R179) were purchased from the National Center for Medical Culture Collections (CMCC, Beijing, China). These clinical isolate strains of *E. coli* (A5), *S.* Pullorum (A2), *S.* Typhimurium (SA59), *S.* Typhimurium (SB323/217), *S.* Typhimurium (SA66/SH286), *S.* Typhimurium (SB209/SH96), *S.* Aureus (A3), and *Proteus mirabilis* (B7) were isolated from clinical samples and maintained by the laboratory of preventive veterinary medicine of Henan Institute of Science and Technology.

Ampicillin, agar powder, Mueller-Hinton broth (MHB), Luria-Bertani broth (LBB), and trypticase soy broth (TSB) medium were purchased from Beijing Solarbio Science & Technology Co., Ltd., Beijing, China. Formaldehyde, glutaraldehyde, and absolute ethanol were of analytical grade.

### Animals

The chickens used in this study were obtained by hatching specific pathogen-free (SPF) chickens embryos in our laboratory. The grouping and treatment of chickens in the experiment are shown in Table 1. This work has received approval for research ethics from LLSC2022014 (Henan Institute of Science and Technology).

**TABLE 1:** Groups of chickens infection test.

Groups	2 h before infection	2 h after infection	Infection of bacteria
G-INF^a^	–	–	8 × 10^5^CFU/mL *Salmonella* Pullorum
G-CON^b^	–	–	No bacteria (PBS)
G-HJH-P^c^	HJH-3: 200 μg/mL IP*, 0.5 mL	–	8 × 10^5^CFU/mL *Salmonella* Pullorum
G-HJH-C^d^	–	HJH-3: 200 μg/mL IP, 0.5 mL	8 × 10^5^CFU/mL *Salmonella* Pullorum
G-Amp-P^e^	Ampicillin: 200 μg/mL IP, 0.5 mL	–	8 × 10^5^CFU/mL *Salmonella* Pullorum
G-Amp-C^f^	–	Ampicillin: 200 μg/mL IP, 0.5 mL	8 × 10^5^CFU/mL *Salmonella* Pullorum

–Nothing done.

^a^The S. Pullorum infection group (G-INF).

^b^The negative control group (G-Con).

^c^The HJH-3 prophylaxis group (G-HJH-P).

^d^The HJH-3 treatment group (G-HJH-C).

^e^The Ampicillin prophylaxis group (G-Amp-P).

^f^The Ampicillin treatment group (G-Amp-C).

*Injected intraperitoneally (IP).

### Physicochemical characterization of HJH-3

The physicochemical characteristics of the AMP HJH-3, including the isoelectric point (PI), liposolubility index, average hydrophobic index and instability index, were analyzed by the Protparam online tool.^1^ The hydrophobicity of the AMP HJH-3 was predicted by the ProtScale online tool.^2^ The structure of HJH-3 was predicted by the I-TASSER method.^3^

### Antimicrobial activity assay *in vitro*

The antimicrobial activities of HJH-3 and Ampicillin were determined by measuring the minimum inhibitory concentrations (MICs) by the twofold broth micro-dilution method according to the CLSI guidance (Humphries et al., 2021). The MIC experiments were repeated three times.

### Time-kill kinetics curve

Solutions of equal parts *S.* Pullorum at a concentration of 5 × 10^5^ CFU/mL in MHB and HJH-3 at different concentrations (0, 50, 100, and 200 μg/mL) were prepared and grown at 37°C. Samples (100 μL) were collected at 0, 1, 2, 4, 6, 8, and 24 h. These samples were diluted 10-fold in LBB. The diluted samples were plated onto LB agar plates and cultured at 37°C for 18 h. The Colony-forming units (CFU) were used to draw the time-kill kinetics curve (Yang et al., 2020). The experiments were repeated three times.

### Hemolytic activity assay

Blood was collected from the chick wing vein and centrifuged at 800 × *g* for 5 min. Then, the supernatant was discarded, and erythrocytes were isolated. The isolated erythrocytes were washed three times with normal saline (0.85%, pH = 7.4), and a suspension of erythrocytes was prepared. HJH-3 (200, 400, 600, or 800 μg/mL) was mixed with an equal volume of a 5% (V/V) erythrocyte suspension, and the mixture was incubated at 37°C for 1 h. Then, the hemolytic activity of HJH-3 was detected by measuring the optical density (OD) of erythrocytes at 414 nm (Norwitz et al., 2005; Wang et al., 2018). Erythrocytes treated with 1% Triton X-100 were used as positive controls (100% hemolysis). Normal erythrocytes without AMP treatment were used as negative controls (no hemolysis). The hemolysis rate of cells was calculated as follows: (OD peptide treatment—OD negative control)/(OD positive control–OD negative control). The experiments were repeated three times.

### Cytotoxicity detection

Chicken embryo fibroblasts (CEFs) were isolated and cultured according to methods reported in the paper (Beug and Graf, 1977). The effect of HJH-3 on the proliferation of CEFs was determined by the CCK-8 method (Yang et al., 2020). HJH-3 and pexiganan (50, 100, 200, and 400 μg/mL) were added to the wells. After incubating the plates for 24 h, 10 μL CCK-8 solutions were added, and the OD at 450 nm was read. Cells without peptides served as positive controls, and medium without cells served as negative controls. Cell viability (%) = [(OD_s_-OD_b_)/(OD_c_-OD_b_)] × 100. ODs = experimental well absorbance (absorbance of wells containing cells, media, CCK-8, and compounds to be tested); OD_b_ = blank well absorbance (absorbance of wells containing medium and CCK-8); OD_c_ = control well absorbance (absorbance of wells containing cells, medium, and CCK-8) (Yang et al., 2020). The experiments were repeated three times.

### LD_50_ detection

The initial concentration of *S.* Pullorum was 6 × 10^9^ CFU/mL. The bacteria were diluted to six gradients, 10^–1^, 10^–2^, 10^–3^, 10^–4^, 10^–5^, and 10^–6^. Forty-two 7-day-old chickens were randomly assigned to seven groups of six chickens each. Among them, six groups were treated with different concentrations of *S.* Pullorum, and 1 group treated with PBS served as the blank control. The challenge dose was 0.5 mL of *S.* Pullorum or sterilized PBS. The challenge was injected intraperitoneally (IP). The morbidity and mortality of chickens were observed after infection. *S.* Pullorum was isolated from diseased chickens, and the data from the chickens were confirmed. The Reed–Muench method was used to calculate the LD_50_ (Haggett and Gunawardena, 1964; Thakur and Fezio, 1981). The calculation process was as follows:

1. Calculate the proportional distance (PD) between the two closest dilutions above and below 50% death: PD = [(% next above 50%)–50%]/[(% next above 50%)–(% next below 50%)];
2. Calculate the 50% end point: Log lower dilution = dilution closest above 50% death;
3. Calculate M by adding PD and Log lower dilution;
4. Calculate LD_50_/mL as 10^–M^/dose.

### Assay of *S.* Pullorum infection

Taking *S.* Pullorum as the research object, the clinical application effect of HJH-3 in the treatment of *S.* Pullorum infection was evaluated in a chicken model. Chicken grouping and infection are shown in Table 1. Sixty chickens were randomly divided into 6 groups: the *S.* Pullorum infection group (G-INF), the HJH-3 prophylaxis group (G-HJH-P), the HJH-3 treatment group (G-HJH-C), the Ampicillin prophylaxis group (G-Amp-P), the Ampicillin treatment group (G-Amp-C), and the negative control group (G-Con). For the prophylaxis group, HJH-3 or Ampicillin was used by IP (200 μg/mL, 0.5 mL), and then the chickens were infected with *S.* Pullorum. For the treatment group, the chickens were infected with *S.* Pullorum, and then HJH-3 or Amp was used by IP.

### Detection of clinical symptoms and internal organ lesions

After initiation of the challenge test, chickens from each group were closely observed for clinical characteristics, such as mental status, coat condition, and fecal characteristics. Clinical symptoms of experimental chickens were divided into three levels after infection with bacteria: Mild symptoms: Lower food intake, fear of cold, body curled up, drooping wings, depression; Severe symptoms: row of white sticky or light yellow or light green loose stool, anus sometimes closed by hardened fecal mass and presence of mild symptoms listed above; Severest symptoms: no food intake, breathing difficulties, and presence of severe symptoms listed above.

After the death of the chickens, they were immediately collected and necropsied on an ultraclean Table 4, and the macroscopic changes in the tissues and organs of all systems of the dead chicks were observed and recorded. Meanwhile, the intestinal contents of dead chickens were aseptically taken to determine the cause of death. The chickens in which no death occurred during the test period were uniformly necropsied after the end of the test and handled according to the method described by Zhang et al. (2015).

### Determination of the bacterial load in the spleen and blood

The agar plate counting method was used to analyze the number of bacteria in the spleen and blood of chickens that died of infection during the experiment as described by Zhang et al. (2015). One hundred microliters of heart-collected blood was directly collected for smearing and counting. The statistical analysis of the spleen bacterial load was performed as follows. Spleens from dead chickens were removed under aseptic conditions (tissues such as the mesangium were removed as much as possible) and weighed. According to the weight of the spleen, a corresponding 20 × volume of sterilized PBS was added. Homogenization of the spleen was performed under sterile conditions, and the homogenization solution was diluted in multiple ratios. Finally, 100 μL extracts were taken for coating and counting. The experiments were repeated three times.

### Pathological observation of internal organs of chickens

The deceased chickens were necropsied, and the internal organs were removed and processed for paraffin sectioning (the approximate procedure was as follows: fixation, dehydration, embedding, sectioning, staining, and blocking) according to a previously reported method (Xu et al., 2012). After that, paraffin sections were placed under a microscope to observe the organ pathology.

### Statistical analysis

Statistical analyses were performed using GraphPad Prism 7.0 (Software). Unpaired Student’s *t*-test or Two-way ANOVA was utilized where appropriate. The threshold of statistical significance level P was set at *P < 0.05, **P < 0.01, ***P < 0.001, and ****P < 0.0001.

## Results

### Physicochemical characterization of the HJH-3

**FIGURE 1:**
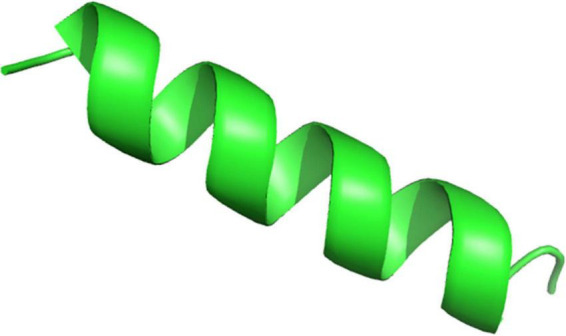
Prediction result of the spatial structure of HJH-3. Copy and paste the sequence of VNFKLLSHSLLVTLRSHL into the Specified location of the website (https://zhanglab.ccmb.med.umich.edu/I-TASSER/). Run the I-TASSER with default settings.

The amino acid sequence of HJH-3 was entered into the website. The physical and chemical properties of HJH-3 are as follows: molecular formula, C_96_H_161_N_27_O_24_; molecular weight, 2077.50 Da; theoretical pI, 11.00; positively charged residues, 2; and instability index of 18.42. The results indicated that HJH-3 is a stable peptide with an estimated half-life of 100 h (mammalian reticulocytes, *in vitro*), > 20 h (yeast, *in vivo*), or >10 h (*E. coli*, *in vivo*). The grand average of hydropathicity (GRAVY) of HJH-3 is 0.70. The results of the prediction of the structure of HJH-3 were shown in Figure 1. Its secondary structure is an α-helix.

### Determination of the antibacterial activity of HJH-3 *in vitro*

The MIC values of HJH-3 against Gram-negative representative bacteria (*E. coli* and *Salmonella*) ranged from 1.5625 to 25 μg/mL. The MIC value for a Gram-positive representative organism (*Staphylococcus*) was greater than 25 μg/mL. HJH-3 also had biological activity against the representative fungus (*C. Albicans*), although the MIC was as high as 100 μg/mL. The results showed that HJH-3 has good antibiotic activity against *Bacillus* Pumilus and *Bacillus* Subtilis. However, HJH-3 had no antibiotic activity against *Actinobacillus* Pleuropneumoniae, *Proteus* Mirabilis, *Pseudomonas* Aeruginosa, or *Enterococcus* Faecalis at a concentration of 200 μg/mL (shown in Table 2).

**TABLE 2:** Minimum inhibitory concentration (MIC) test results of AMP HJH-3 against representative strains.

Strain	Minimum inhibitory concentration (MIC) (μg/mL)	
	HJH-3	Amp
*Salmonella* Pullorum (S. Pullorum CVCC 533)	1.5625	6.25
*S.* Pullorum (A2)^a^	1.5625	6.25
*S.* Choleraesuis (CVCC 3776)	3.125	12.5
*S.* Typhimurium (SA59)^a^	3.125	6.25
*S.* Typhimurium (CVCC541)	6.25	6.25
*S.* Typhimurium (SB323/217)^a^	6.25	100
*S.* Typhimurium (SA66/SH286)^a^	12.5	50
*S.* Typhimurium (SB209/SH96)^a^	25	25
*Escherichia coli* (*E. Coli* (ATCC^®^25922™)	12.5	0.5
*E. coli* (CVCC2059/1568)	25	0.5
*E. coli* (A5)^a^	25	100
*Staphylococcus* Aureus (*S.* Aureus ATCC^®^25923™)	25	0.5
*S.* Aureus (CVCC6538)	50	0.5
*S.* Aureus (A3)^a^	–	–
*Candida* Albicans (ATCC^®^90029™)	100	–
*Bacillus* Pumilus (CMCC63202)	6.25	12.5
*Bacillus* Subtilis (CMCC63501/R179)	12.5	25
*Actinobacillus* Pleuropneumoniae (L20)	–	–
*Proteus* Mirabilis (B7)^a^	–	–
*Pseudomonas* Aeruginosa (CVCC2087)	–	–
Enterococcus Faecalis (R-026)^a^	–	100

^a^The test strains were isolated from clinical samples.

–No antibacterial activity was detected.

### Killing kinetics of HJH-3

**FIGURE 2:**
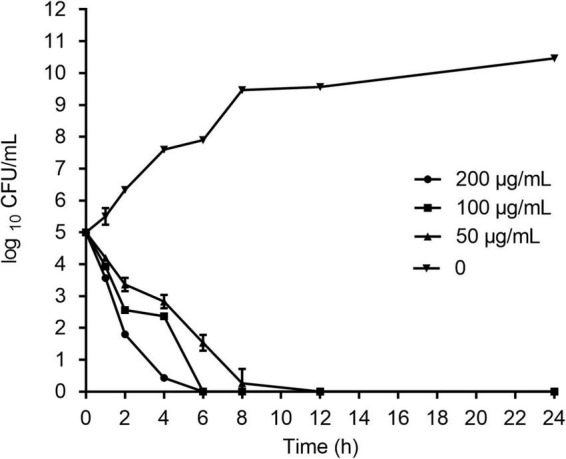
Time-dependent killing curves of *Salmonella* Pullorum incubated with HJH-3. Co-incubation *Salmonella* Pullorum with the different concentrations of HJH-3 (0, 50, 100, and 200 μg/ml) in MH broth medium for 24 h. Aliquots were collected at 0, 1, 2, 4, 6, 8, and 24 h to count the bacteria. Error bars represent means ± SD. The experiments were repeated three times.

As shown in Figure 2, HJH-3 at concentrations of 100 or 200 mg/mL completely killed *S.* Pullorum after incubation for 6 h. HJH-3 at a concentration of 50 mg/mL did not completely kill *S.* Pullorum after co-incubation for 6 h but did completely kill *S.* Pullorum after incubation for 12 h. Therefore, HJH-3 exhibited concentration-dependent bactericidal action against *S.* Pullorum.

### Hemolytic analysis of HJH-3

**FIGURE 3:**
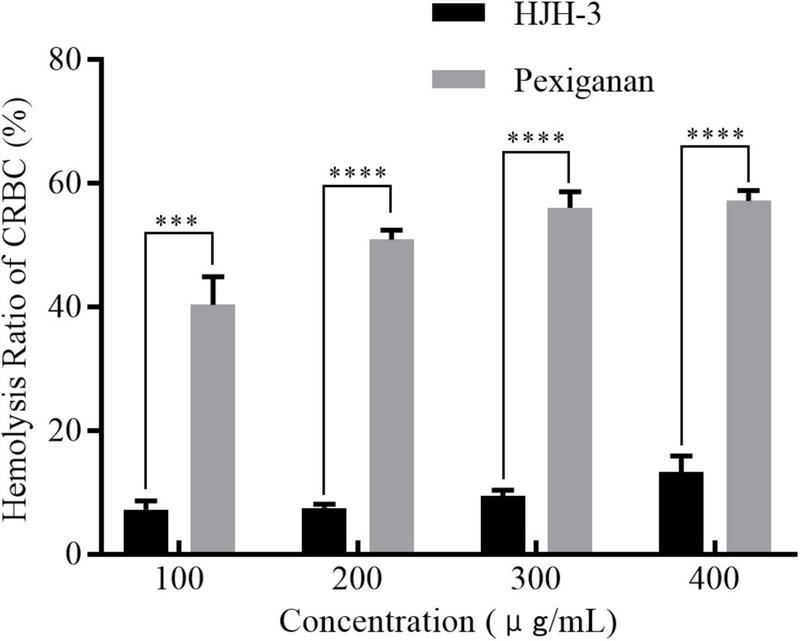
Hemolytic detection of the AMP HJH-3. The isolated erythrocytes were prepared at 5% (V/V) concentration and incubated with HJH-3 (100, 200, 300, or 400 μg/mL) 37°C for 1 h. Then, the hemolytic activity of HJH-3 was detected by measuring the optical density (OD) of erythrocytes at 414 nm. Erythrocytes treated with 1% Triton X-100 were used as positive controls (100% hemolysis). Normal erythrocytes without antimicrobial peptide treatment were used as negative controls (no hemolysis). The hemolysis rate of cells was calculated as follows: (OD peptide treatment—OD negative control)/(OD positive control–OD negative control). The experiments were repeated three times. Results are expressed mean SEM and the statistical analysis was performed using the Unpaired Student’s *t*-test. ***P < 0.001, ****P < 0.0001.

The hemolytic activity of HJH-3 was detected by measuring the absorbance of erythrocytes at OD_414_. The results showed that the hemolysis rate of CRBCs treated with HJH-3 was slightly higher than 10%, even for the 400 μg/mL treatment for 1 h, while the hemolysis rate of the commercial antibacterial peptide pexiganan was as high as 57.3% at 400 μg/mL (Figure 3). Although the two AMPs showed concentration-dependent hemolytic effects, the hemolytic effect of HJH-3 was approximately fourfold lower than that of pexiganan.

### Effect of HJH-3 on CEFs proliferation

**FIGURE 4:**
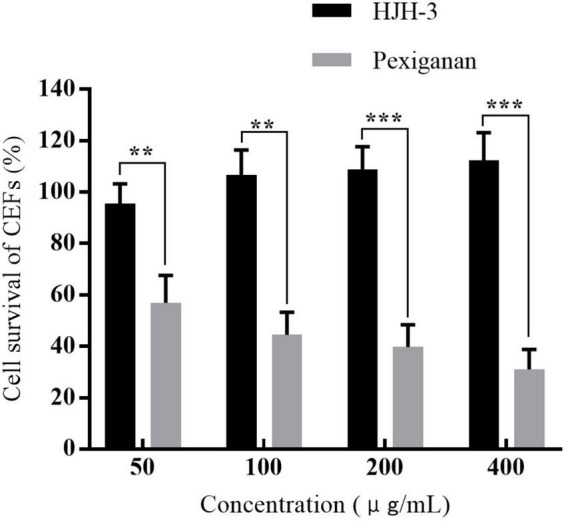
The survival rates of chicken embryo fibroblasts (CEFs) cells treated with HJH-3 and pexiganan (50, 100, 200, and 400 μg/mL) by CCK-8 assay. Results are expressed mean ± SEM and the statistical analysis was performed using the Unpaired Student’s *t*-test. **P < 0.01, ***P < 0.001.

After 24 h of treatment with HJH-3, the CEF survival rate was assessed by CCK-8 assay. The survival rates of CEFs after incubation with HJH-3 at concentrations of 50, 100, 200, and 400 μg/mL were 95.56, 106.65, 108.78, and 112.34%, respectively. The survival rates of CEFs after pexiganan incubation at the same concentrations were 56.93, 43.45, 39.78, and 31.00%, respectively (Figure 4). The CEFs proliferation results showed that cell growth was promoted with increasing HJH-3 concentration and decreased with increasing pexiganan concentration.

### Determination of the LD_50_ in chickens infected with *S.* Pullorum

The mortality of chickens infected with *S.* Pullorum over 7 days was shown in Table 3. The LD_50_ of *S.* Pullorum was calculated according to the calculation process described above. The TCID_50_/0.5 mL was 10^4.375^. The mortality rate of chickens challenged with *S.* Pullorum (6 × 10^9^CFU/mL, diluted by 23713, 0.5 mL, IP) was 50%.

**TABLE 3:** Determination of LD50 in Chickens by *Salmonella* Pullorum.

Group	Bacterial dilution	Total no.	Death no.	Survive no.	Cumulative results			
					Survive	Death	Mortality ratio	Mortality
1	10^–1^	6	6	0	0	29	29/29	100%
2	10^–2^	6	6	0	0	23	23/23	100%
3	10^–3^	6	6	0	0	17	17/17	100%
4	10^–4^	6	5	1	1	11	11/12	91.67%
5	10^–5^	6	4	2	3	6	6/9	66.67%
6	10^–6^	6	2	4	7	2	2/9	22.22%
7*	10^–7^	6	0	6	–			

*Control group.

**TABLE 4:** Groups of chickens infection test.

Groups	Recovered chicken no.	Dead chicken no.	Protection rate (%)	No. of chicken with symptoms		
				Mild symptoms^a^	Severe symptoms^b^	Severest symptom^c^
G-HJH-P	10	0	100	2	0	0
G-HJH-C	8	2	80	2	0	2
G-Amp-P	10	0	100	0	0	0
G-Amp-C	9	1	90	0	0	1
G-INF	0	10	0	0	0	10
G-CON	10	0	100	0	0	0

^a^Mild symptoms: less food, afraid of cold, body curled up, drooping wings, depressed.

^b^Severe symptoms: row of white sticky or light yellow or light green loose stool, anus is sometimes closed by a hardened fecal mass and containing mild symptoms.

^c^Severest symptom: no food, breathing difficulties, and containing severe symptoms.

### Observation of clinical symptoms of chickens infected with *S.* Pullorum

Clinical symptoms in the infected flocks of the different treatment groups were observed for an average of 12 h during the challenge test. The G-INF had the most severe clinical symptoms, including, within 12 h after infection, lowered or no food intake, fear of cold, body curled up, drooping wings, depression, and a preference to aggregate, with death occurring in one chick; on Day 4 after infection, row of white sticky or light yellow or light green loose stool and breathing difficulties; and all 10 chickens infected with S. Pullorum alone dying 6 days after infection. *S.* Pullorum were isolated from the intestinal contents of the dead chicken to ensure that the cause of death was indeed due to infection. The G-HJH-P exhibited no significant clinical symptoms for 3 days after challenge and exhibited mild symptoms in 2 chickens beginning on Day 4, which recovered after 2 days and returned to normal on Day 6. The protection rate was 100%. The G-HJH-C exhibited mild symptoms in 3 chickens on Day 2 post challenge, which recovered by Day 4, and 2 chickens died by Day 5. The protection rate was 80%. In the G-Amp-P, the best treatment effect was observed; no obvious clinical symptoms were observed in infected chicks, and no death occurred in chicks throughout the test period. The protection rate was 100%. In the G-Amp-C, the treatment effect was relatively good; 1 chicken died on the fifth day, and the protective effect was better than that in the AMP treatment group. The G-Con did not show clinical symptoms (shown in Table 4).

### Lesions in the internal organs of chicks after *S.* Pullorum infection

**FIGURE 5:**
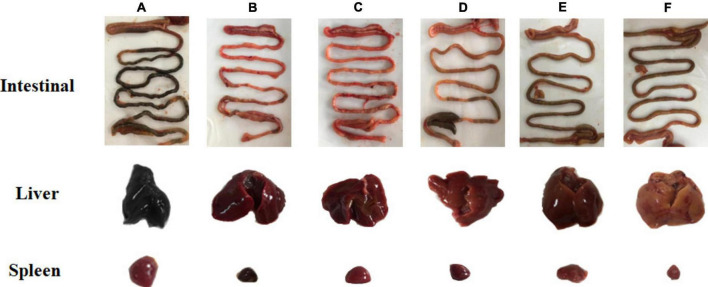
Pathological changes of intestinal, liver, and spleen and in chickens with different treatments. For the prophylaxis group, HJH-3 or Ampicillin was used by injected intraperitoneally (IP) (200 μg/mL, 0.5 mL), and then the chickens were infected with *S.* Pullorum. For the treatment group, the chickens were infected with *S.* Pullorum, and then HJH-3 or Amp was used by IP (200 μg/mL, 0.5 mL). **(A)** Infection group (G-INF); **(B)** antimicrobial peptide treatment group (G-HJH-C); **(C)** antibiotic treatment group (G-Amp-C); **(D)** antimicrobial peptide prevention group (G-HJH-P); **(E)** antibiotic prevention group (G-Amp-P group); **(F)** control group (G-CON).

Chickens challenged with the bacteria were necropsied, and the most severely diseased internal organs were selected for macroscopic analysis of apparent lesions (liver, spleen, intestine, etc.). As shown in Figure 5, lesions in the intestine, spleen, and liver of chicks infected with *S.* Pullorum were prominent. The liver lesions were severe and dark red, and on the cut surface, blood sample foamy exudation was seen. The spleen was grossly enlarged, with a tense tunica and blunt edges. The intestinal lesions were mainly concentrated in the small bowel segments. The duodenum and cecum also had varying degrees of congestion, and the intestinal segments were engorged with ground contents and had a thin and viscous bowel wall. Visceral lesions were milder in the prevention group, and the G-INF exhibited the mildest lesions. Based on the degree of celiac lesions in the dead chickens, the G-HJH-C had a higher degree of lesions than the G-Amp-C.

### Determination of spleen and blood bacterial loads in chicks after *S.* Pullorum infection

**FIGURE 6:**
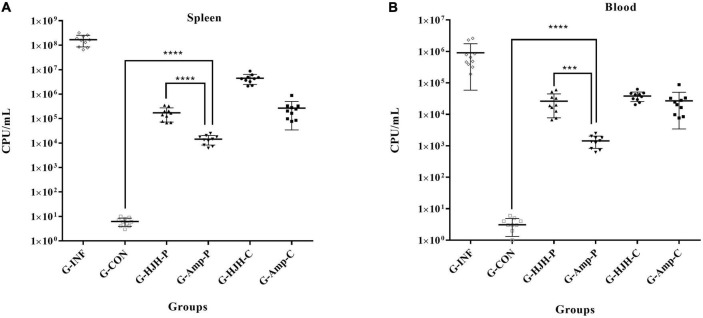
Bacterial load in spleen and blood of different treatment groups. For the prophylaxis group, HJH-3 or Ampicillin was used by injected intraperitoneally (IP) (200 μg/mL, 0.5 mL), and then the chickens were infected with *S.* Pullorum. For the treatment group, the chickens were infected with *S.* Pullorum, and then HJH-3 or Amp was used by IP (200 μg/mL, 0.5 mL). Each point represents data from a single animal, and the line shows the mean value. Results are expressed mean ± SEM and the statistical analysis was performed using the unpaired Student’s *t*-test. ***P < 0.001, ****P < 0.0001. **(A)** Bacterial load in spleen; **(B)** bacterial load in blood.

The total bacterial load in the spleen of G-INF was approximately 1.65 × 10^8^ CFU/g, and the bacterial load in the G-HJH-C was approximately 3.4 × 10^6^ CFU/g, a more than 48-fold decrease. The bacterial loads in the spleens of the chicks in the G-HJH-P and the G-Amp-C were almost the same, approximately 2.0 × 10^5^CFU/g, which was significantly lower than that in the infection alone group. The lowest bacterial load in the spleen of the chicks was observed in the G-Amp-P and was approximately 1.4 × 10^4^ CFU/g (Figure 6A). The bacterial loads in the blood and spleen were largely consistent, but the number of bacteria in the blood was an order of magnitude lower than that in the spleen (Figure 6B).

### Observation of pathological sections of internal organs of chicks after *S.* Pullorum infection

**FIGURE 7:**
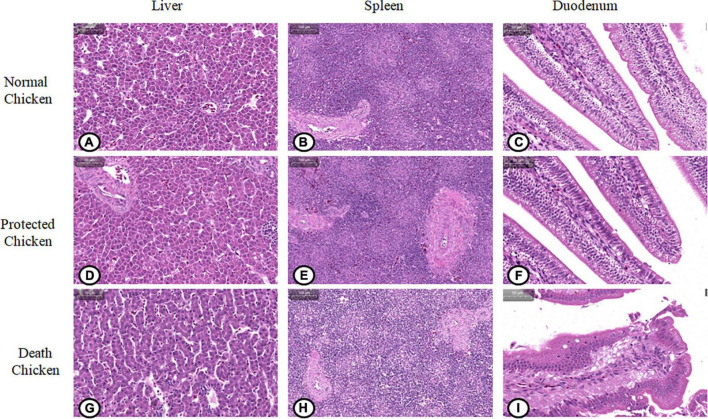
Pathological changes of viscera and organs in chickens infected with *Salmonella* pullorum. **(A)** Liver of normal chickens; **(B)** spleen of normal chickens; **(C)** duodenum of normal chickens; **(D)** liver of protected chickens; **(E)** spleen of protected chickens; **(F)** duodenum of protected chickens; **(G)** liver of chickens with pathological changes; **(H)** spleen of chickens with pathological changes; **(I)** duodenum of chickens with pathological changes.

The paraffin sections in different treatment groups were stained with HE. As shown in Figure 7, the liver lobules of the normal and protected chicks were intact and obvious, the sinusoidal space was obvious, there were detectable lymph nodes and central veins, and there were a few erythrocytes in the liver nucleus (Figures 7A, D). The hepatocytes of chickens infected with *S.* Pullorum were slightly atrophic and smaller, with fatty degeneration and vacuoles appearing, and the number of metachromatic granules increased (Figure 7G).

The normal and protected chicks had a thinner lymphatic sheath around the artery, fewer lymphocytes, an orderly arrangement of lymph nodes, and only a small amount of blood in the spleen sinus (Figures 7B, E). In chicks infected with *S.* Pullorum, the lymphatic sheath around the splenic artery was thickened, the number of lymphocytes was increased, and the number of lymphocytes in the glandular bodies was increased (Figure 7H).

The duodenal glands of normal and protected chicks were intact, the intestinal villi were intact, the striated edges were neat, and there were a few goblet cells in the crypt epithelium (Figures 7C, F). The capillaries of the duodenal lamina propria of chicks infected with *S.* Pullorum were hyperemic and swollen, resulting in congestion. The tops of villi were ruptured, and a large number of villi had sloughed off, which led to the exposure of the lamina propria. The intestinal villi were exfoliated in the intestinal cavity, and the number of goblet cells in the crypt epithelium was increased significantly (Figure 7I).

## Discussion

Antimicrobial peptides have become one of the best substitutes for antibiotics because of their unique antibacterial and non-drug resistance development-inducing characteristics (Sodagari et al., 2020; Ramtahal et al., 2022). Complex eukaryotic organisms AMPs are produced by a variety of host leukocytes and epithelial cells. They are stored in phagocytes, function in lysosomes, or are secreted from cells. Mature AMPs with biological activity are produced after cleavage by endogenous proteases. Human cathelicidin LL-37 originates from hCAP18, which is cleaved by serine proteases (Quintana-Murci, 2006). HJH-3 is a derivative peptide of a natural peptide derived from bovine erythrocyte P3. Sequence alignment showed that included amino acids 97 to 114 of the α-subunit of bovine hemoglobin, which is similar to humans, pigs, deer, and sheep. In contrast to P3, in HJH-3, an A (alanine) was changed to R (arginine), which shifts the net charge to 2, possibly increasing the activity of this AMP. The instability index of HJH-3 is 18.42. It is concluded that HJH-3 is stable and heat-resistant, and these characteristics are consistent with those already described (Wang et al., 2022). Studies have shown that HJH-3 has enhanced antibacterial activity and stability *in vitro* and good application prospects (Zhang et al., 2015; Wang et al., 2022).

In terms of the bactericidal spectrum analysis of HJH-3, this study demonstrated a higher effectiveness against Gram-negative bacteria, especially *Salmonella*, than against Gram-positive bacteria. For clinically isolated drug-resistant strains, HJH-3 had a better antibacterial effect than Ampicillin. Considering the public health problems caused by *S*. Pullorum, the herein described *in vivo* study of HJH-3 against this pathogen is of great relevance.

LD_50_ is the number of bacteria that causes half of the test animals to die, but the number of bacteria that causes death in all animals might not be able to be directly calculated from the LD_50_. Based on the LD_50_ of *S.* Pullorum for the tested 7-day-old chickens and the LD_50_ previously reported (Okamura et al., 2007; Cheng et al., 2020), the number of *S.* Pullorum necessary to cause mortality in 100% of infected chickens was 8 × 10^5^ CFU/mL in our study.

To improve the antibacterial activity of AMPs, a rational determination of AMP administration time can maximize the efficacy of AMPs. Cathelicidin has been used to treat enteritis in mice induced by *Clostridium difficile* 1 day before infection and three consecutive days after infection. It was found that cathelicidin ameliorated the occurrence of intestinal inflammation (Hing et al., 2013). The coprisin can also alleviate diarrhea and colitis caused by bacterial infection in mice by administration in drinking water 1 day before infection and for 6 days after infection (Choi et al., 2016). Regarding the use of AMPs, the choice of administration mode plays a very important role in the prevention and treatment of corresponding diseases (Knappe et al., 2019; Lee et al., 2019; Yu et al., 2020).

Innate immune molecules generally play an important role in the early stage after infection, and based on previously described administration methods (Zhang et al., 2015), this study used the method of one administration early before or after infection to examine the prevention and treatment efficacy of HJH-3 *in vivo*. To ensure the effect of AMPs, different methods and routes of administration were used in this study. The effects of prophylaxis 2 h before infection and treatment 2 h after infection were evaluated separately. The results of animal experiments found that the internal organs showed different degrees of damage, such as thinning of the intestinal tube, congestion of the liver, and splenomegaly, in chickens that appeared symptomatic but did not die. These results suggest that the administration conditions of AMPs still need to be optimized.

The results showed that prophylactic administration of HJH-3 to chickens completely protected against lethal challenge with *S.* Pullorum and reduced bacterial counts in tissue organs and blood and that the overall therapeutic efficacy was consistent with that of antibiotic treatment. It is further indicated that in the clinical applications of AMPs, it is better to use prophylactic administration for disease prevention to fully exert their effects. This study provides a theoretical reference for the further development of HJH-3.

## Conclusion

We investigated the characteristics of the AMP HJH-3, which showed good antibacterial activity and low hemolysis and no cytotoxicity. HJH-3 could completely kill *S.* Pullorum at the concentration of 100 μg/mL after co-incubation for 6 h. Chicken infection model confirmed that HJH-3 could effectively prevent *S.* Pullorum infection by early administration.

## Data availability statement

The original contributions presented in this study are included in the article/supplementary material, further inquiries can be directed to the corresponding authors.

## Ethics statement

The animal study was reviewed and approved by the Laboratory Animal Welfare and Ethical review of Henan Institute of Science and Technology.

## Author contributions

YX and QW: data curation. YX and JH: funding acquisition. MD, HS, BH, and YS: methodology. YS and YX: resources. JH: supervision. All authors contributed to the article and approved the submitted version.

## Footnotes

1
https://web.expasy.org/protparam/

2
https://web.expasy.org/protscale/

3
https://zhanglab.ccmb.med.umich.edu/I-TASSER/
